# Pan-India influenza-like illness (ILI) and Severe acute respiratory infection (SARI) surveillance: epidemiological, clinical and genomic analysis

**DOI:** 10.3389/fpubh.2023.1218292

**Published:** 2023-10-20

**Authors:** Varsha Potdar, Neetu Vijay, Labanya Mukhopadhyay, Neeraj Aggarwal, Sumit Dutt Bhardwaj, Manohar Lal Choudhary, Nivedita Gupta, Harmanmeet Kaur, Jitendra Narayan, Prabhat Kumar, Harpreet Singh, Rizwan Suliankatchi Abdulkader, Manoj Murhekar, Meena Mishra, Sundararajan Thangavel, K. Nagamani, Rahul Dhodapkar, Bashir Ahmad Fomda, Umesh Varshney, Agniva Majumdar, Shanta Dutta, P. Vijayachari, Jyotirmayee Turuk, Tapan Majumdar, Ganesh Chandra Sahoo, Krishna Pandey, Anudita Bhargava, Sanjay Singh Negi, Prabhat K. Khatri, Usha Kalawat, Debasis Biswas, Neeta Khandelwal, Biswajyoti Borkakoty, S. Manjushree, Mini P. Singh, Jyoti Iravane, K. Kaveri, G. B. Shantala, Megha Brijwal, Aashish Choudhary, Lalit Dar, Bharti Malhotra, Amita Jain, ILI-SARI Surveillance Team

**Affiliations:** ^1^ICMR-National Institute of Virology, Pune, India; ^2^Division of Epidemiology and Communicable Diseases, Indian Council of Medical Research, New Delhi, India; ^3^Biomedical Informatics (BMI) Division, Indian Council of Medical Research, New Delhi, India; ^4^ICMR-National Institute of Epidemiology, Chennai, India; ^5^VRDL, All India Institute of Medical Sciences, Nagpur, India; ^6^VRDL, Government Mohan Kumaramangalam Medical College, Salem, India; ^7^VRDL, Gandhi Medical College, Secunderabad, India; ^8^VRDL, Jawaharlal Institute of Postgraduate Medical Education and Research, Puducherry, India; ^9^VRDL, Sher-i-Kashmir Institute of Medical Sciences, Srinagar, India; ^10^VRDL, Government Medical College, Haldwani, India; ^11^ICMR-National Institute of Cholera and Enteric Diseases, Kolkata, India; ^12^ICMR-Regional Medical Research Centre, Port Blair, India; ^13^ICMR-Regional Medical Research Centre, Bhubaneswar, India; ^14^VRDL, Government Medical College, Agartala, India; ^15^ICMR-Rajendra Memorial Research Institute of Medical Sciences, Patna, India; ^16^VRDL, All India Institute of Medical Sciences, Raipur, India; ^17^VRDL, Dr. Sampurnanand Medical College, Jodhpur, India; ^18^VRDL, Sri Venkateswara Institute of Medical Sciences, Tirupati, India; ^19^VRDL, All India Institute of Medical Sciences, Bhopal, India; ^20^VRDL, BJ Medical College, Ahmedabad, India; ^21^ICMR-Regional Medical Research Centre, Dibrugarh, India; ^22^VRDL, Government Medical College, Trivandrum, India; ^23^VRDL, Post Graduate Institute of Medical Education and Research, Chandigarh, India; ^24^VRDL, Government Medical College, Aurangabad, India; ^25^VRDL, King Institute of Preventive Medicine and Research, Chennai, India; ^26^VRDL, Bangalore Medical College and Research Institute, Bangalore, India; ^27^VRDL, All India Institute of Medical Sciences, New Delhi, India; ^28^VRDL, Sawai Man Singh Medical College, Jaipur, India; ^29^VRDL, King George’s Medical University, Lucknow, India

**Keywords:** influenza, integrated surveillance, SARS-CoV-2, SARI, ILI

## Abstract

**Background:**

Over time, COVID-19 testing has significantly declined across the world. However, it is critical to monitor the virus through surveillance. In late 2020, WHO released interim guidance advising the use of the existing Global Influenza Surveillance and Response System (GISRS) for the integrated surveillance of influenza and SARS-CoV-2.

**Methods:**

In July 2021, we initiated a pan-India integrated surveillance for influenza and SARS-CoV-2 through the geographically representative network of Virus Research and Diagnostic Laboratories (VRDLs) across 26 hospital and laboratory sites and 70 community sites. A total of 34,260 cases of influenza-like illness (ILI) and Severe acute respiratory infection (SARI) were enrolled from 4 July 2021 to 31 October 2022.

**Findings:**

Influenza A(H3) and B/Victoria dominated during 2021 monsoon season while A(H1N1)pdm09 dominated during 2022 monsoon season. The SARS-CoV-2 “variants of concern” (VoC) Delta and Omicron predominated in 2021 and 2022, respectively. Increased proportion of SARI was seen in extremes of age: 90% cases in < 1 year; 68% in 1 to 5 years and 61% in ≥ 8  years age group. Approximately 40.7% of enrolled cases only partially fulfilled WHO ILI and SARI case definitions. Influenza- and SARS-CoV-2-infected comorbid patients had higher risks of hospitalization, ICU admission, and oxygen requirement.

**Interpretation:**

The results depicted the varying strains and transmission dynamics of influenza and SARS-CoV-2 viruses over time, thus emphasizing the need to continue and expand surveillance across countries for improved decision making. The study also describes important information related to clinical outcomes of ILI and SARI patients and highlights the need to review existing WHO ILI and SARI case definitions.

## Introduction

The A(H1N1)pdm09 influenza pandemic caused an estimated 0.15–0.58 million deaths in the first year (1), and, in 2020, SARS-CoV-2, caused the coronavirus disease 2019 (COVID-19) pandemic (2). Despite SARS-CoV-2’s high transmissibility and dominance, influenza circulation persisted throughout the COVID-19 pandemic (3). Due to their evolving nature and capability to evade the immune system, the available vaccines for these viruses protect from severe disease but not infection (4, 5).

Given the similarities in the clinical presentation of both viruses and the need to monitor disease trends, WHO released interim guidance in November 2020 (6) recommending the use of the existing Global Influenza Surveillance and Response System (GISRS) for the integrated surveillance of influenza and SARS-CoV-2.

In July 2021, we established country-wide integrated influenza-like illness (ILI) and Severe acute respiratory infection (SARI) surveillance for influenza and SARS-CoV-2 in India in community and hospital settings through the geographically representative network of government-supported Virus Research and Diagnostic Laboratories (VRDLs) (7). The surveillance objectives are: to capture the trends of circulating influenza and SARS-CoV-2 viruses, their types/subtypes/novel strains, the antigenic and genetic diversity of influenza, and monitor antiviral resistance. In addition, the network also captures information on symptomatic illnesses, hospitalizations, and deaths in ILI/SARI patients infected with influenza and/or SARS-CoV-2.

There are only a few global reports of the expansion of existing ILI/SARI surveillance to include the integrated testing of SARS-CoV-2 also (8–10). Here, we present the first comprehensive report of the findings of integrated ILI/SARI surveillance from India.

## Methods

### Site selection, establishment, and organization of the ILI/SARI laboratory network

**Figure 1:**
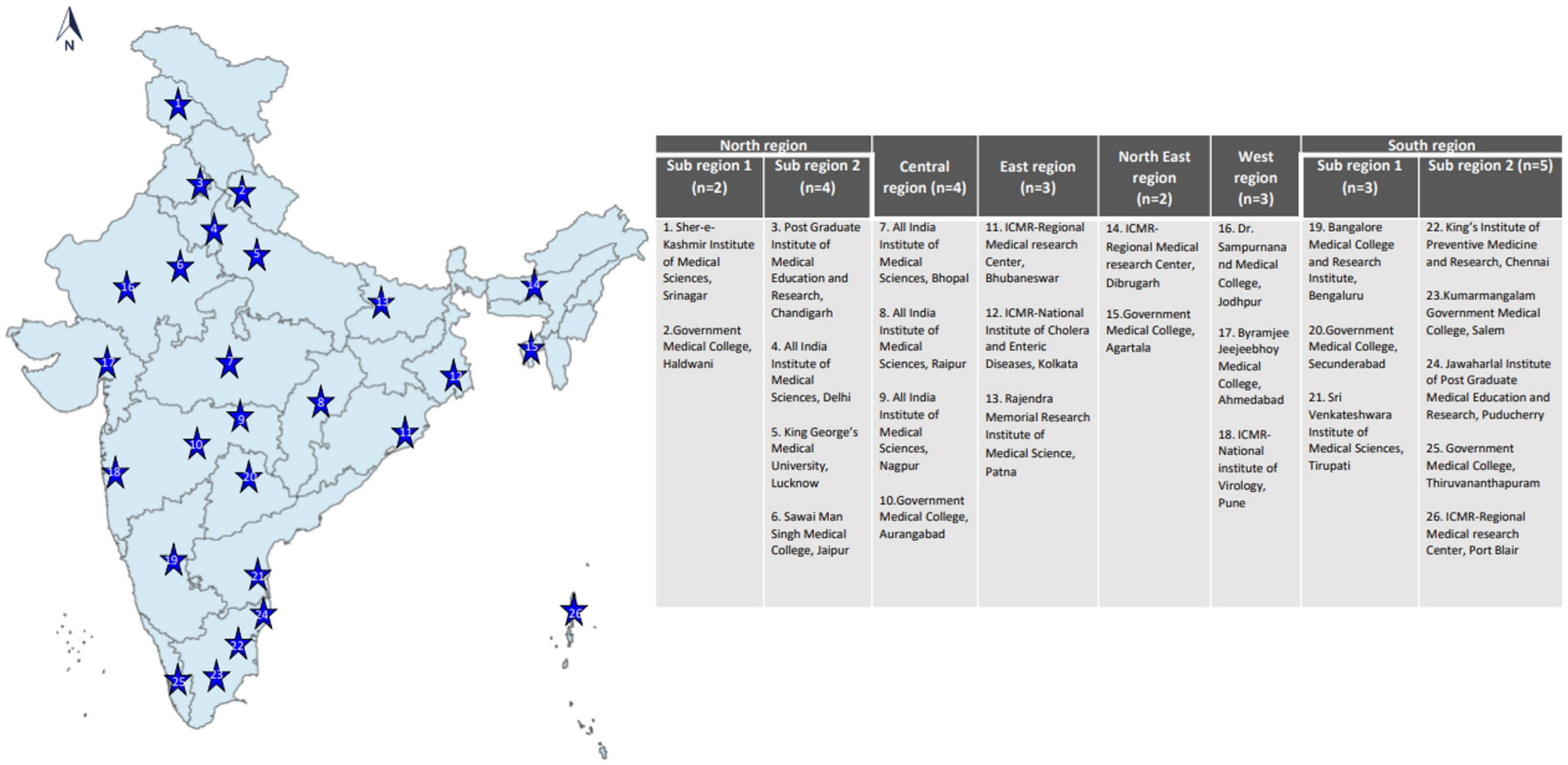
Laboratories (*n* = 26) in the ILI/SARI surveillance network.

In phase 1 (mid-2021), 21 VRDLs were selected (11) and linked with the Indian Council of Medical Research (ICMR)-National Institute of Virology (NIV), Pune, the apex Virology Institute of India, also a WHO-designated National Influenza Center (NIC) and SARS-CoV-2 Referral Centre (21 VRDLs and Indian Council of Medical Research (ICMR)-National Institute of Virology (NIV), Pune brings the total to 22). In phase 2 (mid-2022), four additional laboratories were added. Sites were chosen to ensure a pan-India representation of ILI/SARI cases (Figure 1). Testing data (until October 2022) from 26 sites and clinical data (until June 2022) from 22 sites have been included in this paper. Laboratories were divided into six regions- North, South, East, West, North-East, and Central, and two subregions each in North and South regions. The three-tiered network comprised testing laboratories (TLs: 18), referral laboratories (RLs: 7), and the NIC. All sites undertook enrollment of ILI and SARI patients from community and hospital settings and tested and reported cases every week. RLs also performed isolation and antigenic and genetic characterization of influenza viruses. The NIC undertook training, QA/QC, antigenic and genetic characterization of isolates, drug susceptibility testing, data sharing with WHO FluNet/MART, and provided reagents and technical support to all network laboratories.

### Establishment of surveillance through VRDLs

Each VRDL was linked with relevant clinical departments for enrolling SARI patients. For ILI patients, the primary health system was utilized comprising of subcenters (SCs), primary health centres (PHCs), and community health centres (CHCs), which are the first point of contact for healthcare-seeking in India and represent nearby defined catchment areas. Each VRDL was associated with 3–6 SCs, PHCs, or CHCs. The following WHO case definitions for ILI/SARI and COVID-19 were used (7):

- Influenza-like-illness (ILI): A patient presenting with a reported and measured fever of ≥38°C and cough, with onset within the last 10 days.
- Severe acute respiratory infection (SARI): A patient presenting with a history of fever or measured fever of ≥38°C and cough, with onset within the last 10 days and requiring hospitalization.
- COVID-19: ILI and SARI case definitions were used to identify suspect cases.
- COVID-19/Influenza co-infection: An ILI or SARI case testing positive for SARS-CoV-2 along with influenza A or B virus.
- Influenza A/B dual infection: ILI or SARI case testing positive for influenza A and/or B.

In addition to this, individuals who were already admitted to hospitals due to other causes and later developed SARI were also included in our study.

### Patient selection, sample and data collection, and study duration

Weekly, a minimum of 25 patients (at least 15 ILI and 10 SARI) of all ages were enrolled by each of the participating sites. Clinical pro formas were completed for eligible and consenting patients. Trained personnel collected respiratory specimens (either nasal or throat or nasopharyngeal swabs) in viral transport media (VTM) according to age-appropriate protocols (12). The specimens were transported to the laboratory within 24 h via cold chain (4°C) to ensure the integrity of the samples. In the lab, three aliquots were made for each sample. The first aliquot was used for testing whereas the other two were stored at −80°C.

Data were entered and maintained electronically on a central web portal at the ICMR (13). The testing data presented here are from epidemiological week 27 of 2021 (4 July 2021) to week 43 of 2022 + 2 days (31 October 2022). The clinical data are collated for a 1 year period, from epidemiological week 27 of 2021 (4 July 2021) to week 26 of 2022 + 1 day (3 July 2022).

### Laboratory testing protocol

The NIC developed and validated a multiplex single tube combo real-time reverse transcriptase polymerase chain reaction (rRT-PCR) assay for the simultaneous detection of influenza and SARS-CoV-2 (7). The assay targets were ORF1b of SARS-CoV-2, M1 of influenza A, and NS2 of influenza B, thereby differentiating between influenza A, B, and SARS-CoV-2. The in-house assay was compared with the US-FDA-approved CDC influenza SARS-CoV-2 multiplex assay. For this, 20 influenza A [7-A(H1N1)pdm09 and 13-A(H3N2)], 20 influenza B (10 each of B/Victoria and B/Yamagata lineages), 48 SARS-CoV-2 samples, and 92 clinical samples negative for both influenza and SARS-CoV-2 were simultaneously tested with both the assays. The overall sensitivity and specificity of the in-house assay was 98.9% and 100%, respectively (NIC unpublished data), and it could detect 22 copies of ORF 1b (SARS CoV2), 15 copies of M1 of influenza A, and 12 copies of NS2 of influenza B. Using the in-house assay, the NIC passed WHO influenza and SARS-CoV-2 External Quality Assurance Scheme (EQAS) with 100% concordance. External validation at seven RLs also gave concordant results (unpublished data). Owing to its easy availability, scalability, and economic advantage, the in-house assay was chosen for use in this study.

**Figure 2:**
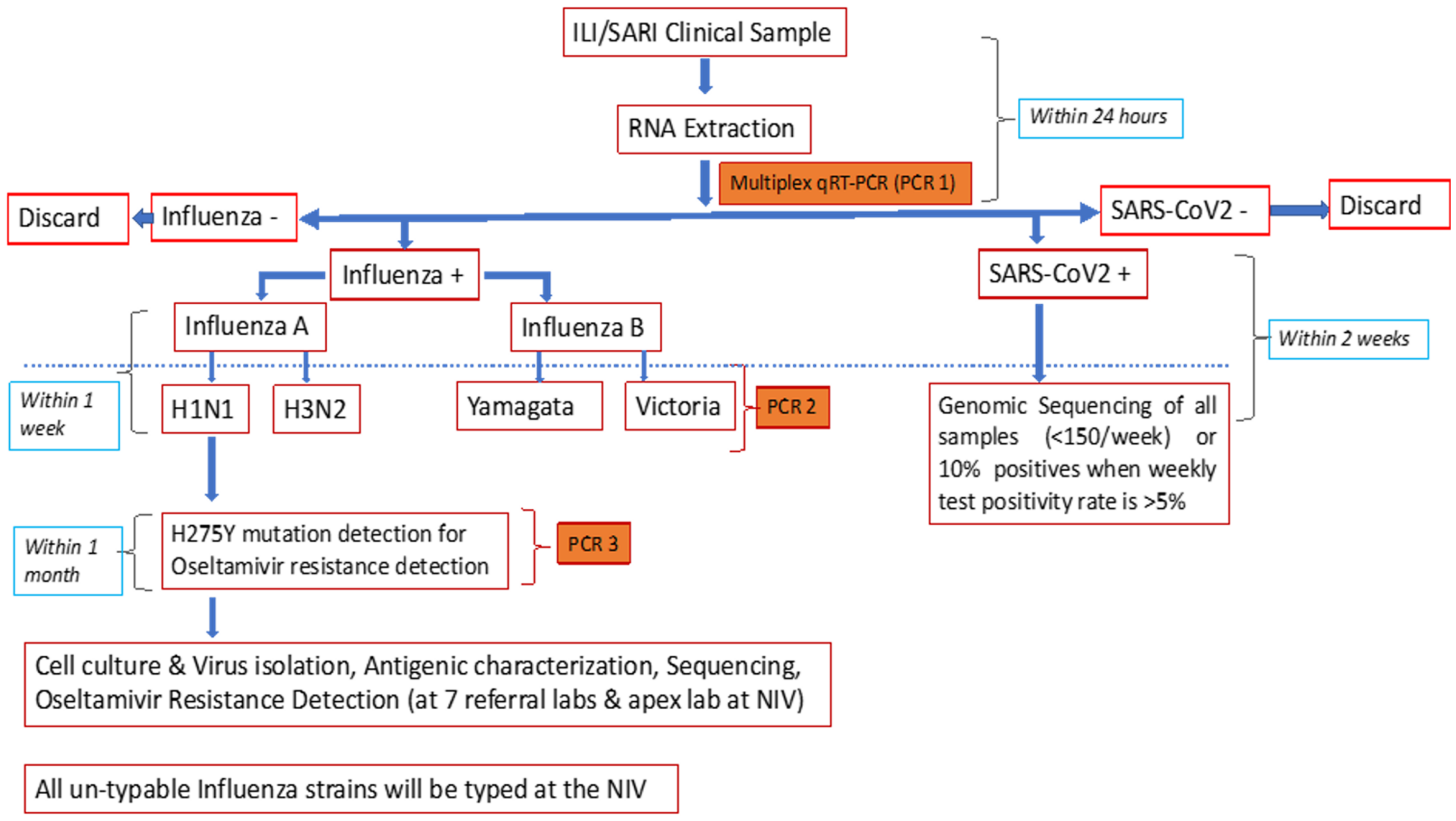
ILI/SARI testing protocol.

Additionally, the CDC subtyping assay was used to differentiate A(H1N1)pdm09 and A(H3) and B/Yamagata and B/Victoria (14). Allelic discrimination assay, using rRT-PCR protocol shared by the National Institute of Health, Thailand (15), was performed to detect H275Y mutations and identify oseltamivir-resistant A(H1N1)pdm09 (Figure 2). All unsubtypable samples were sent to the NIC.

### Training and capacity building of staff and quality control of testing

Staff were trained at the NIC for serological and molecular testing, study protocol/requirements, data collection, and online/offline data entry. Six RLs were also trained for virus isolation, antigenic/ genetic characterization, and sequencing. The NIC obtained a 100% concordance score for influenza and SARS-CoV-2 in External Quality Assurance Programme (EQAP) administered by WHO. The NIC prepared and distributed EQAP panels to all network laboratories before initiating testing (manuscript submitted).

### Influenza virus isolation and characterization

RLs were mandated to perform virus isolation and antigenic characterization using the WHO antigen kit as described by Chadha et al. (16). Hemagglutination (HA) and hemagglutination inhibition (HI) tests confirmed virus isolation and identified influenza virus subtypes. Representative isolates were submitted to WHO Collaborating Centres in Atlanta, United States, and Melbourne, Australia (17).

### Sequencing and phylogenetic analysis of influenza virus

Briefly, HA gene sequencing was done in a subset of positive samples using an ABI 3730 DNA analyzer as described earlier (16, 18). Sequences were curated by Seqscape V2.5 software (Applied Biosystems, United States). Pairwise sequence alignment and phylogeny of the HA gene were performed using best fit Tamura-Nei nucleotide substitution model to generate a neighbor-joining tree. The MEGA 6 program (19) was used to generate multiple sequence alignment and phylogenetic trees. SARS-CoV-2 positive samples were referred to INSACOG laboratories for sequencing. The trends of prevailing variants of SARS-CoV-2 at different time points in India can be accessed on the Indian SARS-CoV-2 Genomics Consortium (INSACOG) dashboard (20).

### Statistical analysis

All data were entered into an Excel sheet and analyzed using SPSS Version 21.0. The chi-square test was used to calculate the odds ratio (OR), and it represented the association between potential exposures and different outcomes. The age-adjusted odds ratio was calculated using the logistic regression model for assessing the risk of ICU admission and death among patients who tested positive for influenza A(H1N1)pdm09 and A(H3N2), influenza B (B/Victoria and B/Yamagata), and SARS-CoV-2. For this, the dependent variables were ICU admission and death of patients (no = 0 and yes = 1) while the independent variables were virus types that were assigned zero if the virus strain was not present and 1 if present. *p* < 0.001 was considered to indicate the level of significance.

## Results

### ILI/SARI test results

A total of 34,260 [20,105 ILI (58.7%) and 14,155 SARI (41.3%)] cases were enrolled and tested between 4 July 2021 and 31 October 2022 with 10, 20, 26, 21, and 23% of cases enrolled from the Central, East, North, South, and West regions, respectively. In total, 1,261 (6.2%) and 1,172 (5.9%) influenza and SARS-CoV-2 infections belonged to the ILI group, respectively, while 1,297 (9.2%) and 709 (5%) influenza and SARS-CoV-2 infections belonged to the SARI group, respectively (Table 1). Of the 4,439 positive samples, 37 were dual/co-infections (33 influenza/COVID-19 co-infections and 4 influenza A/B dual infections). A total of 4,402 (12.8%) individuals were positive for influenza and/or SARS-CoV-2.

**Table 1:** Age-wise distribution and positivity of ILI and SARI patients.

Age group	Total patients tested (%)	ILI			SARI		
		Cases^a^	Positive for influenza^b^	Positive for SARS-CoV-2^b^	Cases^a^	Positive for influenza^b^	Positive for SARS-CoV-2^b^
<1 year	2,780 (8.1%)	290 (10%)	17 (5.9%)	11 (3.8%)	2,490 (90%)	101 (4.1%)	86 (3.5%)
1 to 5 years	3,470 (10.1%)	1,120 (32%)	134 (12%)	22 (2%)	2,350 (68%)	240 (10.2%)	57 (2.4%)
5 to 18 years	4,472 (13.1%)	2,733 (61%)	285 (10.4%)	88 (3.2%)	1,739 (39%)	219 (12.6%)	63 (3.6%)
18 to 45 years	13,829 (40.4%)	10,647 (77%)	589 (5.5%)	663 (6.2%)	3,182 (23%)	331 (10.4%)	224 (7%)
45 to 60 years	4,820 (14.1%)	2,925 (61%)	142 (4.9%)	210 (7.2%)	1,895 (39%)	188 (9.9%)	119 (6.3%)
60 to 80 years	4,364 (12.7%)	2,180 (50%)	86 (3.9%)	156 (7.2%)	2,184 (50%)	189(8.6%)	137 (6.3%)
≥80 years	490 (1.4%)	191 (39%)	7 (3.7%)	21 (11%)	299 (61%)	29 (9.7%)	20 (6.7%)
Missing age details	35 (0.1%)	19 (54.3%)	1 (2.8%)	1 (2.8%)	16 (45.7%)	0	3 (8.57%)
Total	34,260	20,105 (58.7%)	1,261 (6.2%)	1,172 (5.8%)	14,155 (41.3%)	1,297 (9.1%)	709 (5%)

a Percentages are calculated on the basis of total number of patients tested in that age group.

b Percentages are calculated as: (number of cases positive in a particular age group/total number of ILI or SARI cases screened in that age group) × 100.

### Distribution of viruses

**Figure 3:**
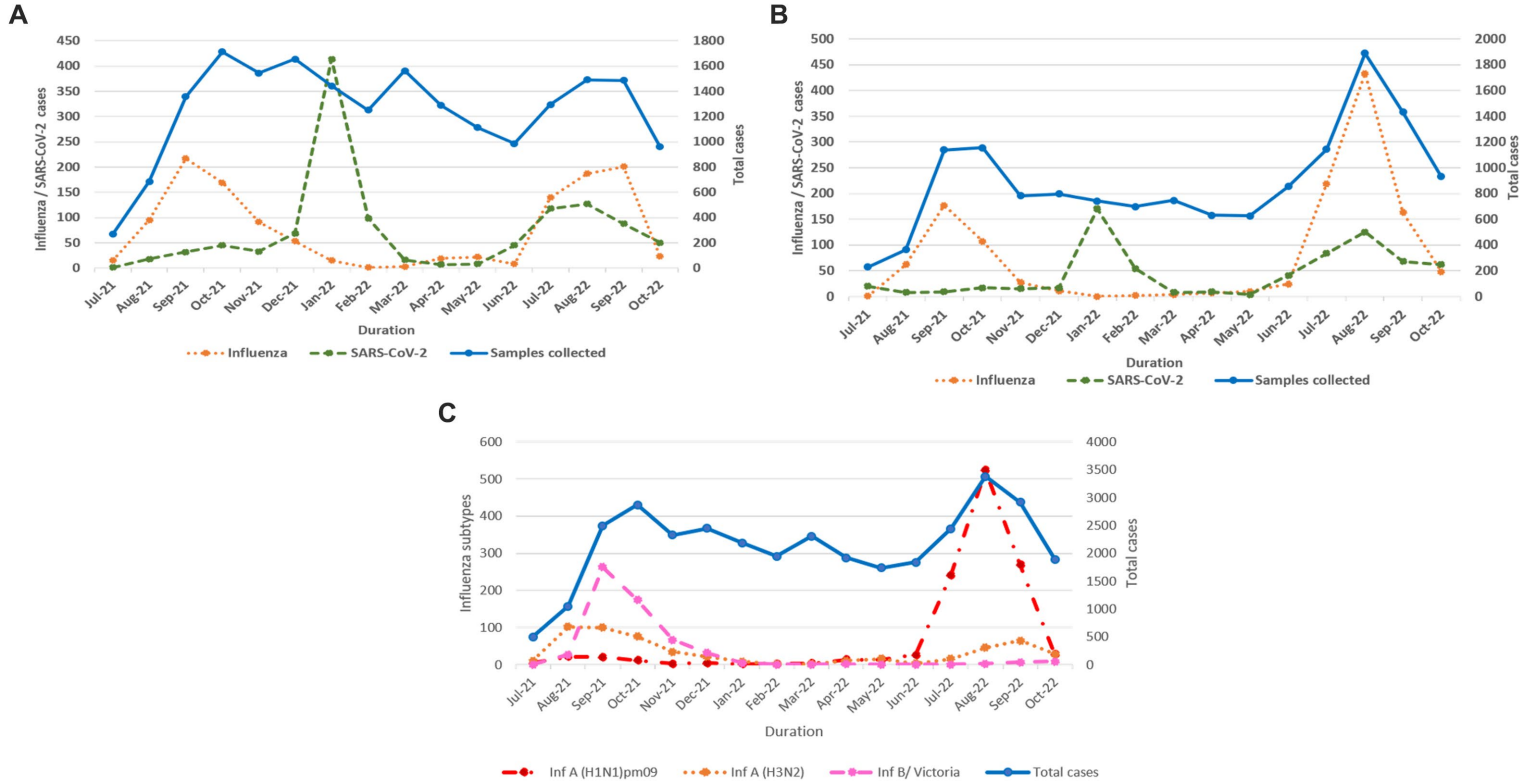
**(A)** Monthly distribution of influenza and SARS-CoV-2 detected among ILI cases. **(B)** Monthly distribution of influenza and SARS-CoV-2 detected among SARI cases. **(C)** Monthly distribution of influenza virus sub types.

Influenza and SARS-CoV-2 positivity trends varied throughout the study period (Figures 3A,B). In 2021, overall influenza positivity rose from mid-July (3.2%) to a peak in September (15.8%) followed by steady decline. In 2022, influenza circulation was low until May followed by a sharp rise from mid-June, a peak in August (18.4%), and then a gradual decline. SARS-CoV-2 positivity in 2021 remained low (1.6% to 4.2%) before rising sharply to 26.7% in January 2022 followed by a decline before peaking again to 7.5% positivity in August 2022.

### Influenza virus subtypes and distribution in 2021 and 2022

**Figure 4:**
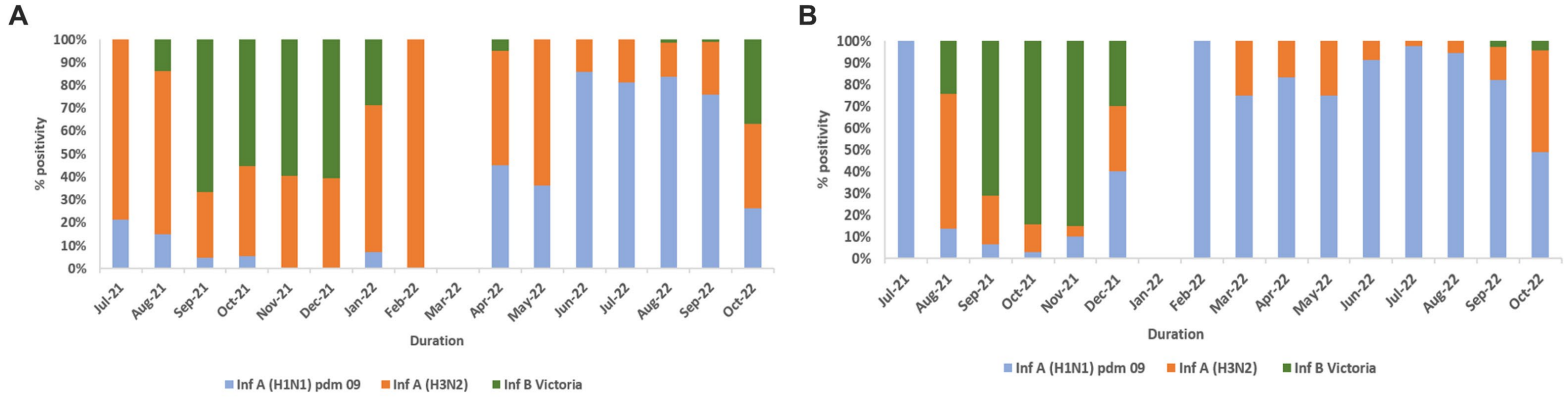
**(A)** Influenza virus subtype wise % positivity among ILI cases. **(B)** Influenza virus subtype wise % positivity among SARI cases.

In total, 2,317 of 2,558 influenza-positive samples could be subtyped. The remaining samples could not be subtyped due to low quantity. A(H1N1)pdm09, A(H3N2), and B/Victoria positivity was 1,189 (51.3%), 543 (23.4%), and 585 (25.2%), respectively (Figure 3C). The distribution of subtypes within ILI and SARI is shown in Figures 4A,B. Among ILI cases, 380 (34.7%), 372 (34%) and 340 (31.1%) infections were of A(H1N1)pdm09, A(H3N2) and B/Victoria respectively. Among SARI cases, 809 (66%), 171 (14%) and 245 (20%) infections were of A(H1N1)pdm09, A(H3N2) and B/Victoria respectively. No untypable A, B, or Yamagata strains were reported. The circulation of subtypes varied throughout the study (Figure 3C). In 2021, influenza B circulation was significant, whereas in 2022 influenza A was higher. B/Victoria (66.6%) and A(H3N2) predominated during the peak influenza activity of September 2021, while A(H1N1)pdm09 predominated (84.3%) in the second peak of August 2022. Type B circulation decreased in 2022, with <10 cases/month from January to October and no recorded cases in February, May–July, and September 2022. All but three (all from Pune, India) influenza A(H1N1)pdm09 isolates collected and tested in this period were sensitive to the neuraminidase inhibitor, Oseltamivir. These three isolates had H275Y mutations in the NA gene as determined by allelic discrimination rRT-PCR. Two of these three isolates were successfully isolated in cell culture and had reduced susceptibility to Oseltamivir in phenotypic assay.

### Distribution of SARS-CoV-2 and influenza cases across six regions and two sub-regions

**Figure 5:**
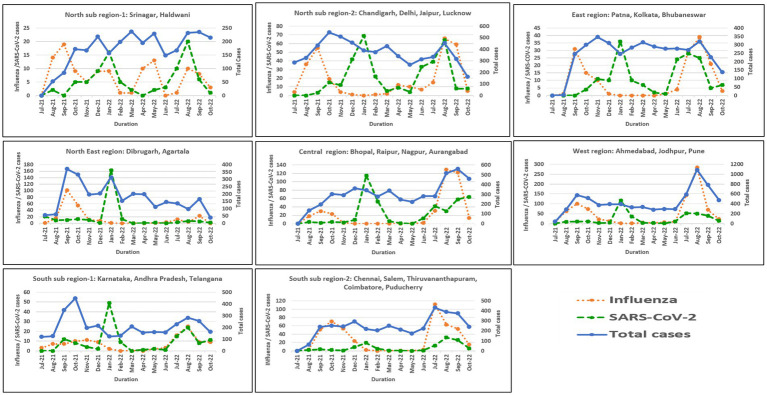
Regional trends of influenza and SARS-CoV-2 positivity across India.

In North India, both sub-regions showed peak influenza activity in September 2021 and August–September 2022. However, an additional peak was observed in sub-region 1 in May 2022. Northeast, East, Central, West, and South (sub-region 1) showed similar trends with peak influenza activity in September 2021 and August–September 2022. However, sub-region 2 of South India showed a sustained peak in October–November 2021 and July–September 2022. All six regions showed COVID-19 peaks during the Omicron wave in India in January 2022. Another relatively smaller peak was seen in August 2022 in East, Northeast, and North India, in August–September in South India, September–October 2022 in Central India, and July–August 2022 in West India. Regional influenza and SARS-CoV-2 positivity is depicted in Figure 5.

### Sex and ILI/SARI positivity

Of the 34,260 cases, sex demographics were unavailable for 25 cases. In total, 19,530 (57%) cases were males and 14,705 (43%) were females. The distribution of ILI and SARI was 56% and 44% for males and 58 and 42% for females, respectively. Test positivity in males and females was comparable, i.e., 13% and 12.6%, respectively, with equal influenza positivity of 7.5% and 7.4%, respectively. The distribution of influenza-positive males and females in ILI was 3.7% and 3.8% respectively while in SARI it was 3.7% each. SARS-CoV-2 positivity in the ILI and SARI groups was 3.4% and 2.2% for males and 3.4% and 1.9% for females, respectively.

### Age-stratified analysis

Patients were split into seven age groups (<1 year, ≥1 to <5 years, ≥5 to <18 years, ≥18 to <45 years, ≥45 to <60 years, ≥60 to <80 years, and ≥80 years) for analysis (Table 1). Age information was available for 34,225/34,260 patients. The age group with the most patients was the ≥18 to <45 years group (40.4%). 61%, 77% and 61% cases of ILI were seen in 5 - <18 years; 18 ± <45 years and 45 -<60 years respectively. 39%, 23% and 39% cases of SARI were seen in 5 - <18 years; 18 ± <45 years and 45 -<60 years respectively. Equal proportion of ILI and SARI patients fell in the ≥60 to <80 years group. It is noteworthy that in the extreme age groups, <1 year, ≥1 to <5 years, and ≥80 years, ILI proportion was low, i.e., 10%, 32%, and 39%, respectively, with the majority presenting as SARI: 90%, 68%, and 61%, respectively.

### ILI and SARI cases outside WHO case definition

Of the 34,260 cases, 20,307 (59.3%) were enrolled as per WHO case definitions of ILI and SARI. A total of 40.7% cases (13,953/34,260) did not fully satisfy the WHO case definitions. However, influenza and SARS-CoV-2 positivity was reported in such cases with 1,528/4,402 (34.7%) positive samples falling in this category: 659, 231, and 648 cases were positive for influenza A, B, and SARS-CoV-2, respectively. Ten cases had COVID-19/influenza co-infection.

### Proportion of individuals with comorbidities

In total, 4,556/34,260 (13.3%) patients had one or more comorbidities (Table 2). Individuals with chronic pulmonary diseases, diabetes mellitus, haematological disorders, heart diseases, chronic hepatic and renal diseases, malignancy, obesity, hypertension, HIV, and patients on various immunosuppressant therapies were considered comorbid. The proportion of SARI was higher (68.5%) than ILI (31.5%) in this group.

**Table 2:** Distribution of ILI and SARI comorbid patients.

Age group	Total screened (%)	ILI				SARI			
		Cases^a^	Positive for influenza^a^	Positive for SARS-CoV-2^a^	Co-infections	Cases^b^	Positive for influenza^b^	Positive for SARS-CoV-2^b^	Co-infections
<1 year	201 (4.4%)	10 (5%)	0	0	0	191 (95%)	5 (2.6%)	3 (1.6%)	0
1 to 5 years	267 (5.9%)	21 (7.9%)	1 (4.8%)	0	0	246 (92.1 %)	18 (7.3%)	3 (1.2%)	1 (0.4%)
5 to 18 years	344 (7.6%)	85 (24.7%)	4 (4.7%)	7 (8.2%)	0	259 (75.3%)	27 (10.4%)	12 (4.6%)	0
18 to 45 years	1,087 (23.9%)	538 (49.5%)	19 (3.5%)	24 (4.5%)	0	549 (50.5%)	52 (9.5%)	32 (5.8%)	1 (0.2%)
45 to 60 years	1,051 (23.1%)	364 (34.6%)	16 (4.4%)	25 (6.9%)	0	687 (65.4%)	82 (11.9%)	37 (5.1%)	1 (0.1%)
60 to 80 years	1,388 (30.5%)	374 (26.9%)	17 (4%)	20 (5.3%)	1 (0.3%)	1,014 (73.1%)	111 (10.9%)	52 (5.1%)	1 (0.1%)
≥80 years	211 (4.6%)	42 (19.9%)	0	4 (9.5%)	0	169 (80.1%)	18 (10.7%)	11 (6.5%)	0
Missing age details	7 (0.2%)	2 (28.6%)	1 (50%)	1 (50%)	0	5 (71.4%)	0	1 (20%)	0
Total	4,556	1,436 (31.5%)	58 (4%)	81 (5.6%)	1 (0.1%)	3,120 (68.5%)	313 (10%)	151 (4.8%)	4 (0.1%)

a Percentages are calculated on the basis of total number of patients tested in that age group.

b Percentages are calculated as: (number of cases positive in a particular age group/total number of ILI or SARI cases screened in that age group) × 100.

### Influenza virus isolation and genetic characterization

**Figure 6:**
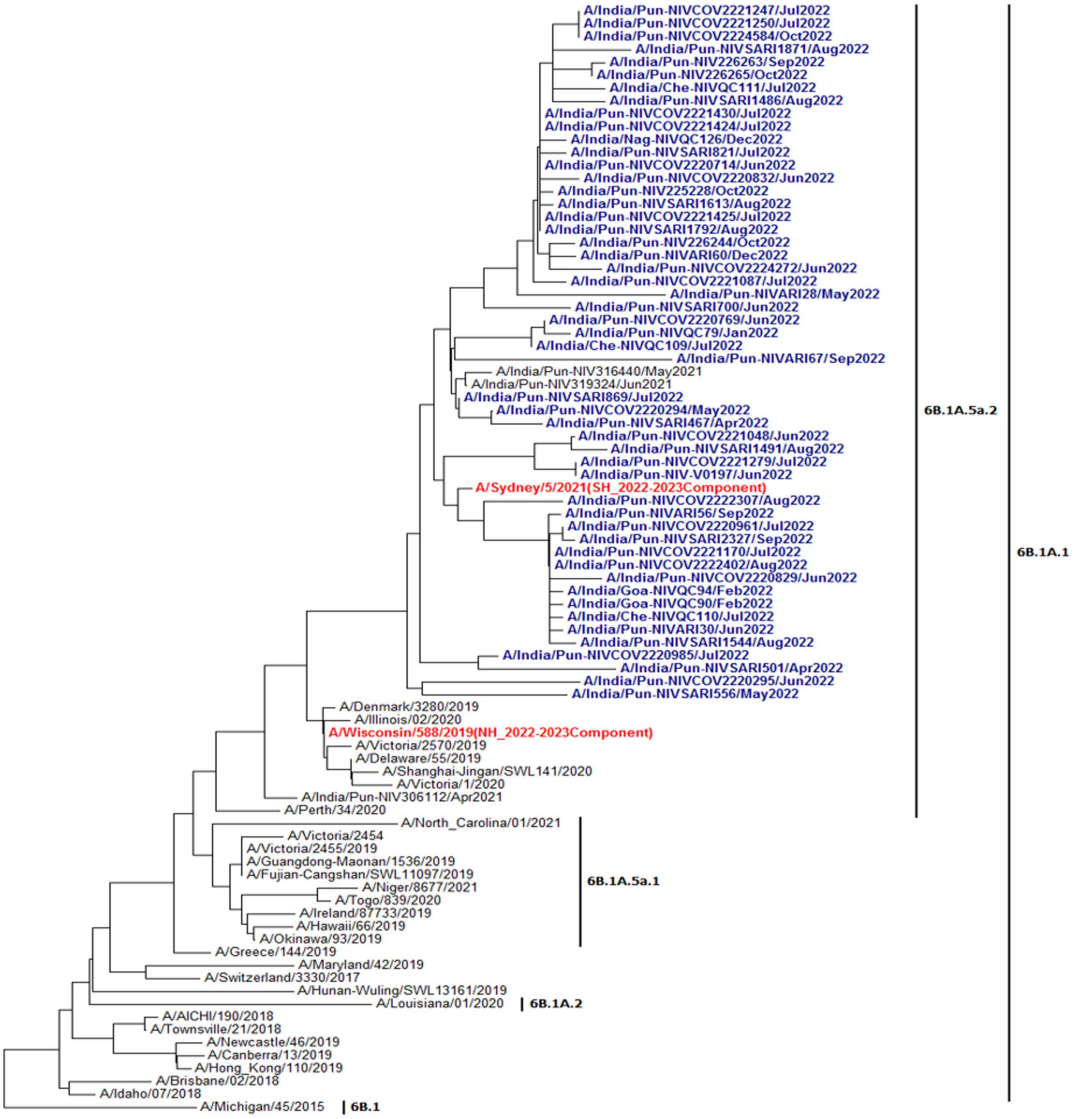

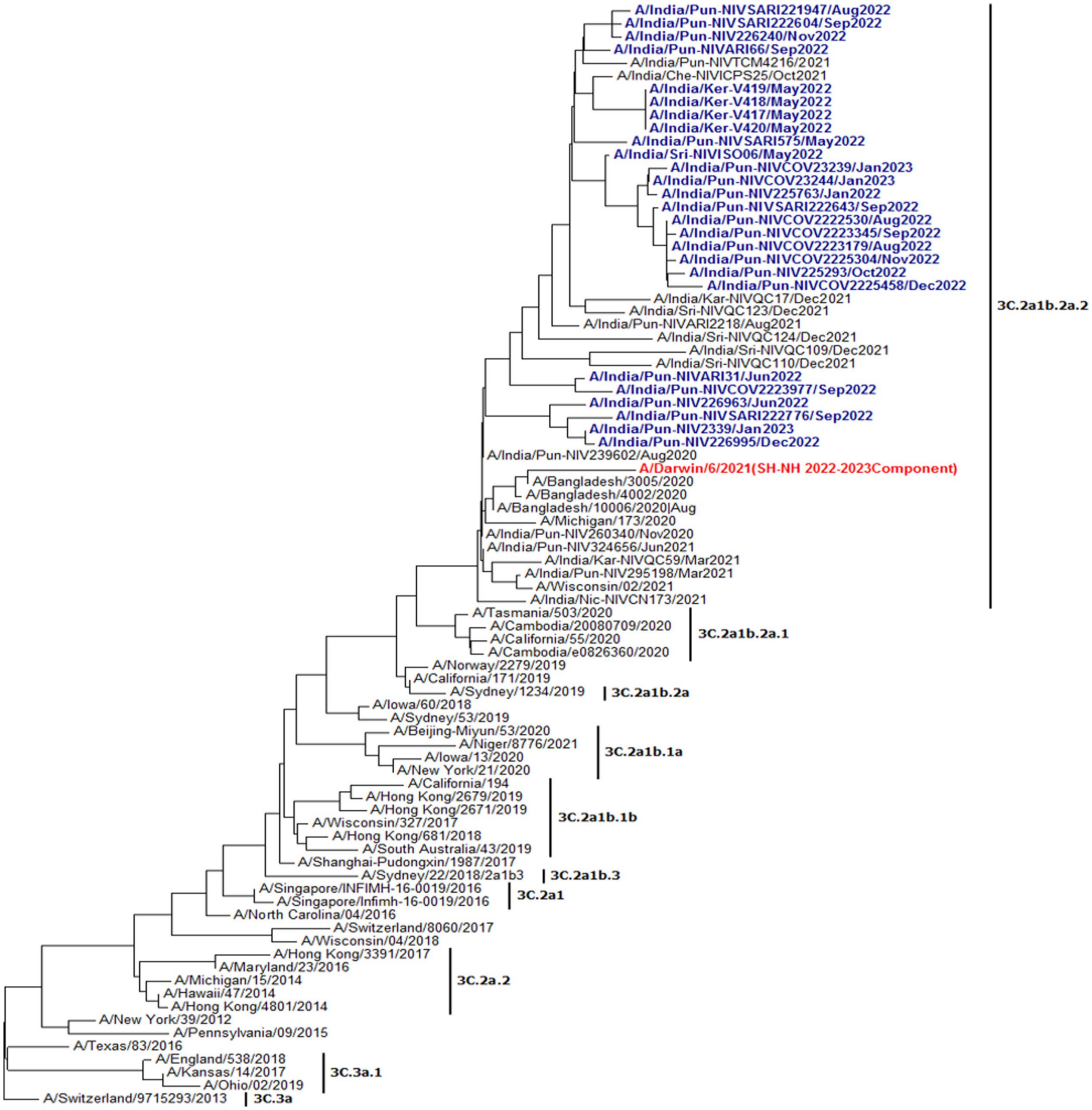

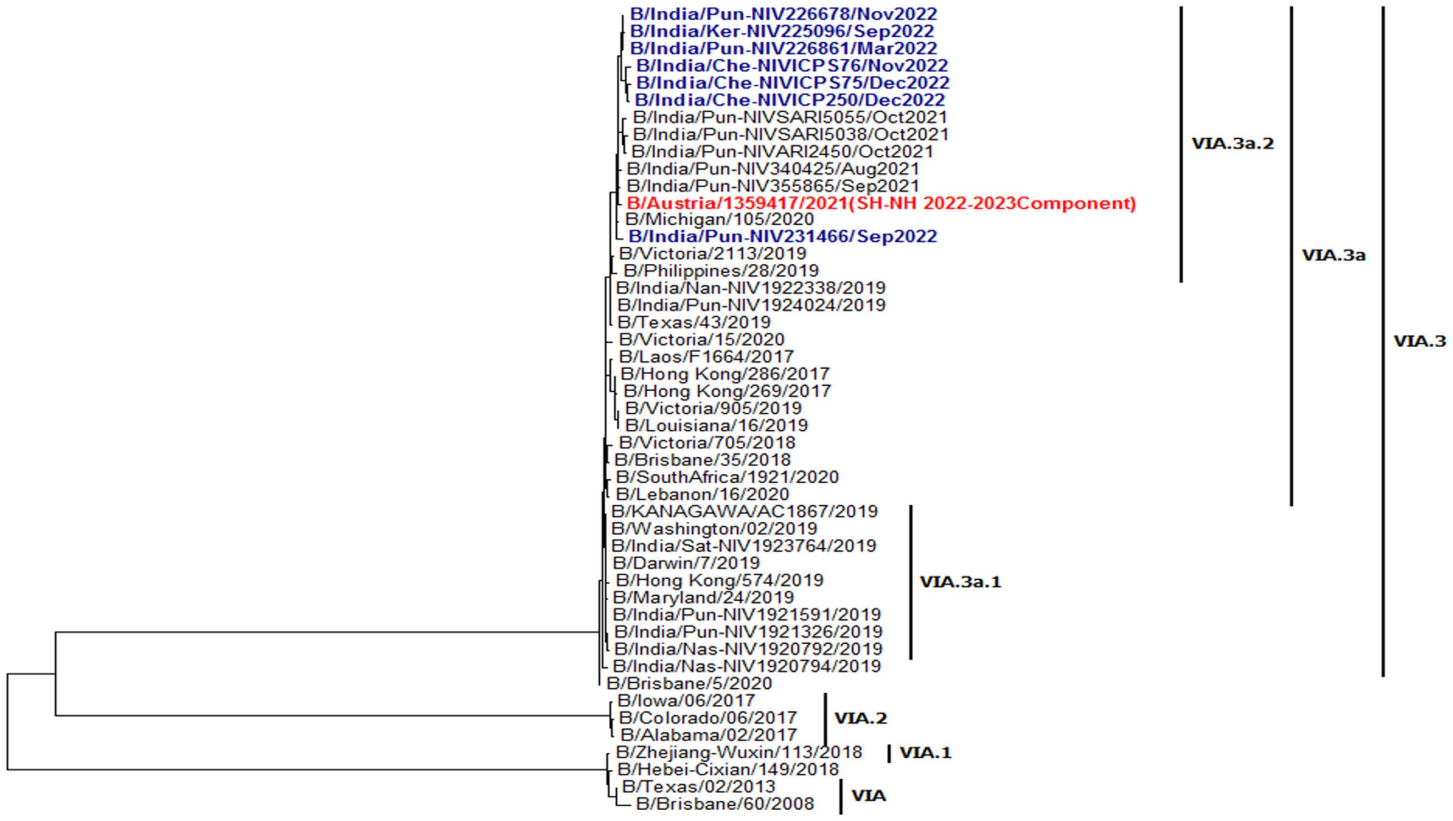
Phylogenetic analysis of A(H1Nl)pdm09 from 2021 and 2022. The strains in red are 2022–23 vaccine component strains. The 2022 strains are denoted in blue. Phylocenetic analysis of A(H3N2) from 2021 and 2022. The strain in red is 2022–23 vaccine component strains. The 2022 strains are denoted in blue. Phylogenetic analysis of influenza B viruses: B/Victoria lineage from 2021 and 2022. The strain in red is 2022–23 vaccine component strains. The 2022 strains are denoted in blue.

Virus isolation in the Madin-Darby Canine Kidney (MDCK) cell line was undertaken in 898 influenza-positive (295 A(H1N1)pdm09, 395 A(H3N2), and 208 B/Victoria) samples spread across the study period. Isolation of 241 viruses (135 A(H1N1)pdm09, 67 A(H3N2), and 39 B/Victoria) was confirmed by HA and further subtyping by HAI tests. HA gene sequencing was performed in 136 isolates by Sanger method and 73 A(H1N1)pdm09, 38 A(H3N2), and 25 B/Victoria were sequenced. Phylogenetic trees were constructed using representative study strains of all three viruses and are presented in Figures 6A–C. A(H1N1)pdm09 virus strains belonged to 6B.1A.1A subclade 5a.2, the globally predominant circulating strain with HA1 substitutions: K130N, N156K, L161I, and V250A. Subclade 5a.2 is similar to A/Victoria/2570/2019-like and A/Sydney/5/2021-like viruses, representing the vaccine viruses for the 2022–2023 northern hemisphere and 2023 southern hemisphere influenza seasons, respectively (Figure 6). A(H3N2) virus strains belonged to 3C.2a1b subclade 2a.2 with HA1 substitutions: Y159N, T160I, L164Q, G186D, D190N, F193S, and Y195F. Antigenically, the A(H3N2) strains belonged to the 2a.2 subclade similar to A/Darwin/6/2021-like viruses, the vaccine strains for the 2022–2023 northern hemisphere and the 2023 southern hemisphere influenza seasons (Figure 6). Influenza B/Victoria/2/87 strains belonged to clade V1A.3 with amino acid deletion of three residues (162–164) in HA1 and a K136E HA1 substitution. Strains from subclade V1A.3a.2 [HA1 substitutions of A127T, P144L, N150K, G184E, and N197D (resulting in the loss of N-linked glycosylation motif, K203R, and R297K)] were predominant. Antigenic analysis showed that 2021 and 2022 strains in subclade 3a.2 were similar to B/Austria/1359417/2021-like viruses (subclade 3a.2), representing the vaccine viruses for the 2022–2023 northern hemisphere and the 2023 southern hemisphere influenza seasons (Figure 6).

### Clinical outcomes of patients

**Figure 7:**
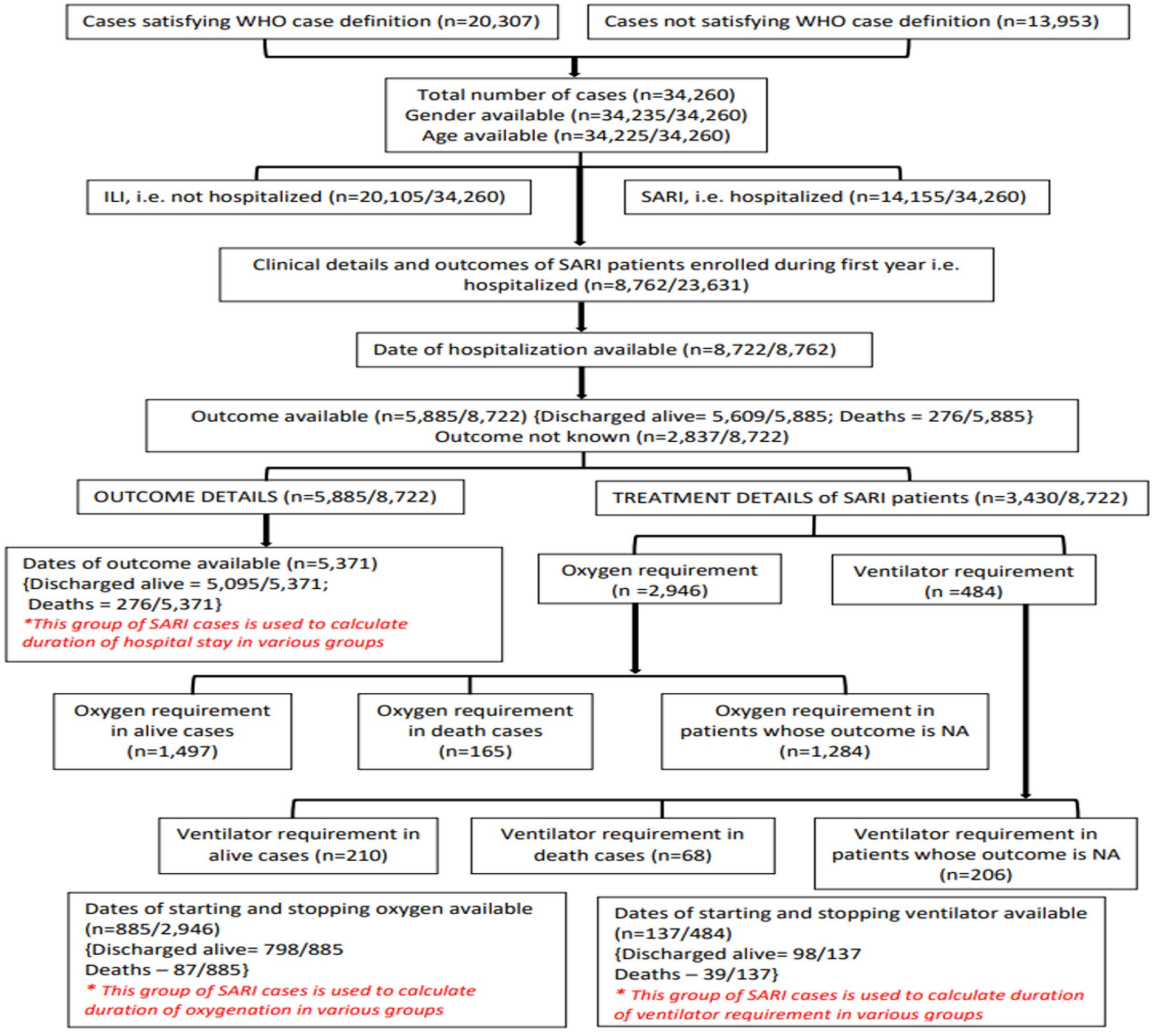
Flowchart depicting different subsets of patients used for analysis of clinical data.

Of the 23,631 patients enrolled in the first year, 8,762 were SARI cases. Dates of hospitalization were available for 8,722 patients. Details of the various SARI outcomes analyzed are given in Figure 7. The results are summarized in Tables 3, 4.

**Table 3:** Clinical details of 23,631 patients (enrolled from 4 July 2021 to 3 July 2022).

S. No.	Parameter	Total	Positives	Influenza	SARS-CoV-2	Co-infection
1	Patients hospitalized as per the availability of date of hospitalization (date of hospitalization was not available for 40 patients)	8,722 (37%)	788 (9%)	420 (53.3%)	370 (47%)	2
1a	Patients with comorbidities	1,850 (21.2%)	153 (8.3%)	84 (54.9%)	69 (45%)	0
1b	Patients with no comorbidity	6,912 (79.2%)	647 (9.4%)	348 (53.8%)	301 (46.5%)	2
1c	ICU admission	2,056 (23.6%)	203 (9.9%)	120 (59.1%)	84 (41.4%)	1
1d	Oxygen required	2,946 (33.8%)	227 (7.7%)	122 (53.7%)	105 (46.3%)	0
1e	On ventilator	484 (5.5%)	42 (8.6%)	20 (47.6%)	22 (52.4%)	0
2	**Outcome of hospitalized patients**					
2.1	Discharged alive (date of discharge available for 5,095 patients)	5,609 (64.3%)	463 (8.3%)	228 (49.2%)	235 (50.8%)	0
2.1.1	Median duration of hospitalization of 5,095 cases discharged alive	7 days				
2.1.2	Oxygen requirement	1,497 (26.7%)	98 (6.5%)	53 (54.1%)	45 (45.9%)	0
2.1.3	Median duration of oxygenation in 798 cases discharged alive (based on available data of start and weaning dates)	5 days				
2.1.4	In ICU	840 (15%)	67 (8%)	37 (55.2%)	30 (44.8%)	0
2.1.5	On ventilator	210 (3.7%)	11 (5.2%)	4 (36.4%)	7 (63.6%)	0
2.1.6	Median duration of ventilation in 98 cases discharged alive (based on available data of start and weaning dates)	4 days				
2.2	Deaths	276 (3.2%)	24 (8.7%)	11 (45.8%)	13 (54.2%)	0
2.2.1	Median duration in hospital until death of 276 cases	9 days				
2.2.2	Oxygen requirement	165 (59.8%)	15 (9.1%)	7 (46.7%)	8 (53.3%)	0
2.2.3	In ICU	117 (42.4%)	17 (14.5%)	5 (29.4%)	12 (70.6%)	0
2.2.4	On ventilator	68 (24.6%)	11 (16.2%)	4 (36.4%)	7 (63.6%)	0
2.2.5	Median duration of oxygenation in 87 deaths (based on available data of start and weaning dates)	8 days				
2.2.6	Median duration of ventilation in 39 deaths (based on available data of start and weaning dates)	6 days				
2.3	Outcome not available	2,837 (32.5%)	301 (10.6%)	181 (60.1%)	122 (40.5%)	2

**Table 4:** Odds ratios in influenza- and SARS-CoV-2-infected and co-infected patients versus SARI patients negative for both the viruses.

S. No.	Variables	SARS-CoV-2 and/or influenza			Influenza			SARS-CoV-2		
		Odds ratio	95% CI	*p*-value	Odds ratio	95% CI	*p*-value	Odds ratio	95% CI	*p*-value
**In all patients**										
1	Hospitalization	0.9	0.8 to 1	0.02	1	0.9 to 1.2	0.69	0.8	0.7 to 0.9	0.00
2	ICU admission	1.1	1 to 1.3	0.13	1.3	1 to 1.6	0.01	1	0.8 to 1	0.77
3	Oxygen requirement	0.8	0.7 to 0.9	0.0008	0.7	0.6 to 0.9	0.01	0.7	0.6 to 1	0.02
4	Ventilator	0.9	0.7 to 1.3	0.72	0.8	0.5 to 1.3	0.42	1.1	0.7 to 1.7	0.74
**In comorbid patients**										
5	Hospitalization	3.5	3.2 to 3.8	<0.0001^*^	4.4	3 to 6.42	<0.0001^*^	3	2 to 4.3	<0.0001^*^
6	ICU admission	2.2	1.6 to 3.1	<0.0001^*^	1.6	1 to 2.6	0.05	3.2	2 to 5.2	<0.0001^*^
7	Oxygen requirement	1.6	1.5 to 1.8	<0.0001^*^	3.7	2.5 to 5.4	<0.0001^*^	2.4	1.6 to 3.7	0.0001^*^
8	Ventilator requirement	1.3	0.6 to 2.8	0.44	0.2	0.03 to 1.7	0.14	2.2	1 to 4.7	0.057
**In pregnant women**										
9	Hospitalization	3.3	1.2 to 8.9	0.02						
**Death in patients**										
10	Non-comorbid	1.1	0.7 to 1.6	0.795	1	0.5 to 1.8	1	1.1	0.6 to 2	0.677
11	Comorbid	1.6	1.2 to 2.1	0.001^*^						

^*^Significant *p*-value.

### Odds ratios

- The odds of hospitalization, oxygen requirement, ICU admission, and ventilator need in influenza- and SARS-CoV-2-positive patients (combined and separate analysis) was similar to those testing negative for both viruses. Only influenza-infected females had 1.5 times increased odds of ICU admission. Other subgroups were insignificant.
- When compared to non-comorbid positive patients, the odds of hospitalization, ICU admissions, and oxygen requirement in comorbid patients positive for both viruses (influenza and SARS-CoV-2) together was 3.5, 2.2, and 1.6 (*p* < 0.0001), respectively. For SARS-CoV-2 alone, the odds were 3, 3.2, and 2.4 (*p* < 0.0001) for hospitalization, ICU admission, and oxygen requirement, respectively. For influenza, the odds were 4.4 (*p* < 0.0001) for hospitalization and 3.7 for oxygen requirement. The remaining subgroups were either insignificant or the numbers were too small for analysis.
- The odds of hospitalization in influenza- and SARS-CoV-2-positive pregnant women (combined analysis) were found to be non-significant. Since the numbers were small, subgroup analysis for influenza- and SARS-CoV-2-positive patients could not be undertaken. The outcome was known for 42 pregnant SARI cases (out of 46): all were discharged alive.
- Odds of death of patients positive for Influenza and/or SARS-CoV-2 was not different from those testing negative for both viruses. However, the odds ratio was 1.6 (*p* = 0.001) for the co-morbid patients positive for influenza or SARS-CoV-2 (Table 4).
- Overall, age was not associated with the increased risks of ICU admission and death for A(H1N1)pdm09, A(H3N2), B/Victoria, and SARS CoV-2 infections. However, when the adjusted odds ratio was calculated for different age groups, it was found that SARS-CoV-2-infected individuals aged 45–59 years had 3.2 times higher likelihood of ICU admission (Table 5). No association was seen with increased risk of mortality when split by different ages.

**Table 5:** Adjusted odds ratio for ICU admission among different age groups.

Hospitalized patients below 18 years	Adj. OR	(95% CI)	*p*-value
Inf A(H1N1)pdm09	1.5	(0.3, 7.4)	0.625
Inf A(H3N2)	2.0	(0.7, 5.8)	0.202
Inf B/Victoria	1.5	(1, 2.5)	0.082
SARS-CoV-2	0.6	(0.3, 1.3)	0.162

Hospitalized patients between 18–44 years	Adj. OR	(95% CI)	*p*-value
Inf A(H1N1)pdm09	3.2	(0.4, 29)	0.36
Inf A(H3N2)	0.4	(0.1, 2.8)	0.264
Inf B/Victoria	0.4	(0.1, 2.8)	0.264
SARS-CoV-2	0.7	(0.2, 1.9)	0.43

Hospitalized patients between 45–59 years	Adj. OR	(95% CI)	*p*-value
Inf A(H1N1)pdm09	2.8	(0.7, 10.9)	0.173
Inf A(H3N2)	1.1	(0.1, 9)	0.941
Inf B/Victoria	1.1	(0.2, 4.9)	0.917
SARS-CoV-2	3.2	(1.8, 5.6)	<0.001^*^

Hospitalized patients 60 years and above	Adj. OR	(95% CI)	*p*-value
Inf A(H1N1)pdm09	4.4	(1.5, 12.6)	0.009
Inf A(H3N2)	0	(0, 1.171e+301)	0.116
Inf B/Victoria	1.9	(0.6, 6.4)	0.296
SARS-CoV-2	1.2	(0.6, 2.2)	0.65

^*^Significant *p*-value. The reference groups are patients that tested negative for respective influenza and/or SARS-CoV-2 viruses, e.g., a patient negative for A(H3N2) is coded as 0 and taken as reference group against those who are positive for A(H3N2) and thus were given a value of 1.

### Outcomes and duration of hospitalization

Outcomes were available for 5,885/8,762 (67.2%) SARI cases. In total, 5,609/5,885 (95.3%) patients were discharged alive and 276/5,885 (4.7%) patients died. The median duration of hospitalization was 7 days for both males and females who were discharged alive and 9 and 10 days in females and males who died, respectively. The proportion of deaths in different age groups, <1 year, ≥1 to <5 years, ≥5 to <18 years, ≥18 to <45 years, ≥45 to <60 years, ≥60 to <80 years, and ≥80 years were 9, 5, 6, 2, 4, 6, and 5%, respectively. The proportion of individuals discharged alive ranged from 91% to 98% in different age groups.

## Discussion

India has a significant burden of acute viral respiratory infections across ages (21). Sporadic studies have been published on the proportion of respiratory syncytial virus (RSV)-related illness in children under 5 (22) and influenza in adults (23). However, systematic ILI/SARI surveillance to continuously capture trends of prevailing strains of influenza and SARS-CoV-2 has been missing. The geographically representative pan-India ILI/SARI surveillance network established by us captures the trends of respiratory viruses from community and hospital settings. We found that influenza A(H3N2) and B/Victoria predominated during the 2021 monsoon season, followed by a decline which persisted throughout winter (December 2021 to February 2022). This coincided with the Omicron wave of COVID-19 in India. Until the summer of 2022, we observed that when influenza transmission peaked, SARS-CoV-2 circulation declined and vice versa. However, during the 2022 influenza monsoon peak, we documented an upsurge of both viruses together, with 7.5% and 18.6% positivity of SARS-CoV-2 and influenza in August 2022, respectively. The change in transmission dynamics of the viruses may be attributed to the difference in circulating strains. In 2021, influenza A(H3N2) and the Delta variant of concern (VoC) of SARS-CoV-2 were predominant, whereas influenza A(H1N1)pdm09 and the Omicron VoC of SARS-CoV-2 predominated in 2022.

In an earlier study, distinct influenza seasonality was reported across India (16) over a 5 year period. Influenza peaks were seen in August–September in North India, June–July in Northeast and East India, July–September in Central and Western India, and September–November in South India. In our study, matching peak influenza activity was seen in North India (subregion 2); whereas in Northeast, East, Central, and Western India, the peak shifted from June–July to August–September. In South India (subregion 2), sustained peaks in October–November 2021 and July–September 2022 were seen, in contrast to an earlier study. Our study documented few differences in the timings of peak influenza activity as compared with the earlier report. Since the seasonal trends help in understanding the appropriate timings of annual influenza vaccinations, it is necessary to monitor the seasonality and timing of influenza outbreaks in different parts of India on a continued basis.

We observed age-stratified differences in disease severity. Similar to other studies (24, 25), an increased proportion of SARI was documented in the extremes of age, with 90% cases in <1 year, 68% in ≥1 to <5 years, and 61% in ≥80 years, with overall influenza positivity of 4.2%, 11%, and 7.3%, respectively. Influenza vaccine uptake during the past 1 year in our study was only 0.3%. Influenza vaccination in India is available only in the private sector on a voluntary basis. Our findings reiterate the need for influenza vaccinations in younger (<5 years) and older (>80 years) age groups (26). Low influenza and SARS-CoV-2 positivity in the <1 year age group suggests the need to test for other pathogens, especially respiratory syncytial virus (RSV) and relevant bacterial infections, for instituting appropriate clinical management protocols.

Our data show a virus isolation rate of about 33%, which is comparable to other reports for oral and nasal samples (27). Successful isolation in cell culture is highly dependent on the quality of the sample, i.e., the titer of live virus in the sample. Though we resorted to using samples with high viral titers (based on low cycle threshold (Ct) values in rRT-PCR) but degradation during storage or transport cannot be ruled out. The susceptibility of cells to influenza viruses could also influence the rate of virus isolation, and certain cell lines derived from parent MDCK cells have been shown to have higher susceptibility for influenza viruses (28, 29). However, parent MDCK cells continue to be the preferred cell line for primary isolation of the influenza viruses (30) and thus was the cell line of choice in this study.

Phylogenetic analysis of multiple isolates indicated that A(H1N1)pdm09, A(H3N2), and B/Victoria lineages matched with globally circulating influenza strains. Further, the Indian strains matched with the vaccine virus strains recommended by WHO for the 2022–2023 northern hemisphere and 2023 southern hemisphere influenza seasons (31).

ILI/SARI case definitions by WHO are uniformly used across the world. We also used the same and trained the staff accordingly. However, 40.7% of the recruited cases and 34.7% of the positives only partially fulfilled the WHO case definitions. Previous studies have analyzed the sensitivity and specificity of WHO case definitions for influenza detection (32, 33). Our findings suggest that a significant number of cases infected with influenza and SARS-CoV-2 are missed by strictly following the WHO case definitions. The definitions need to be reviewed, taking into account global data.

As per the available literature (34), we also found that influenza- and COVID-19-infected comorbid patients and pregnant women were more prone to hospitalization, ICU admission, and ventilator support. We did not document sex disparities in disease severity except that influenza-infected females were predisposed to ICU admission.

Our study has a few limitations. Firstly, follow-ups and clinical outcomes were only available in a small subset. Secondly, we could only include two respiratory viruses in our surveillance. The inclusion of respiratory syncytial virus (RSV) in the paediatric population would have yielded more comprehensive information. Thirdly, we could only isolate and characterize a small subset of influenza viruses, and antigenic characterization for influenza vaccine selection could not be done. However, overall, our ongoing ILI and SARI surveillance system has yielded valuable information on the trends of circulating influenza and SARS-CoV-2 strains and their molecular characteristics and clinical outcomes in patients in an ethnically and genetically diverse country like India. In line with the global goal of integrated surveillance of respiratory viruses (35), we have set up a geographically representative network of ILI and SARI surveillance in India. Moving ahead, it is important to sustain this network and expand surveillance to include other respiratory viruses and also strengthen the network to detect novel strains of influenza emerging at animal-human interfaces (36) from a One Health perspective.

## Data availability statement

The original contributions presented in the study are included in the article/Supplementary material, further inquiries can be directed to the corresponding author/s.

## Ethics statement

The studies involving humans were approved by Institutional Review Board of all the surveillance sites and ICMR-NIV, Pune, India. The studies were conducted in accordance with the local legislation and institutional requirements. Written informed consent for participation in this study was provided by the participants' legal guardians/next of kin.

## Author contributions

VP: standardization and validation of the testing methods, training, quality testing, isolation, and resources. NV: project administration and coordination with sites, funding acquisition, data curation, formal analysis, and writing—original draft. LM: data curation, formal analysis, and writing—original draft. NA: project administration, funding acquisition, data curation, formal analysis, review, and finalization of the paper. SB: methodology, resources, and validation. MC: investigation, methodology, resources, and validation. NG conceptualized the whole plan of integrated surveillance through the network of VRDLs, critical writing, data review, and finalization of the paper. HK: data analysis and referencing. JN: coordination with the surveillance sites. PKu and HS: software development and management. SR: implmentation of the project at four sites in Tamil Nadu. MaM: coordination and review of the Tamil Nadu sites. MeM (PI*, VRDL, All India Institute of Medical Sciences Nagpur), TS (PI*, VRDL, Government Mohan Kumaramangalam Medical College, Salem), KN (PI*, VRDL, Government Medical College Secunderabad), RD (PI*, VRDL, Jawaharlal Institute of Postgraduate Medical Education and Research, Puducherry), BF (PI*, VRDL, Sher-i-Kashmir Institute of Medical Sciences, Srinagar), UV (PI*, VRDL, Government Medical College, Haldwani), SD and AM (PI*, ICMR-National Institute of Cholera and Enteric Diseases, Kolkata), PV (PI*, ICMR-Regional Medical Research Centre, Port Blair), JT (PI*, ICMR-Regional Medical Research Centre, Bhubaneswar), TM (PI*, VRDL, Government Medical College, Agartala), KP and GS (PI*, ICMR-Rajendra Memorial Research Institute of Medical Sciences, Patna), SN and AB (PI*, VRDL, All India Institute of Medical Sciences, Raipur), PKh (PI*, VRDL, Dr. Sampurnanand Medical College, Jodhpur), UK (PI*, VRDL, Sri Venkateswara Institute of Medical Sciences, Tirupati), DB (PI*, VRDL, All India Institute of Medical Sciences Bhopal), NK (PI*, VRDL, BJ Medical College, Ahmedabad), BB (PI*, ICMR-Regional Medical Research Centre, Dibrugarh), SM (PI*, VRDL, Government Medical College, Trivandram), MPS (PI*, VRDL, Post Graduate Institute of Medical Education and Research, Chandigarh), JI (PI*, VRDL, Government Medical College, Aurangabad), KK (PI*, VRDL, King Institute of Preventive Medicine and Research, Chennai), GS (PI*, VRDL, Bangalore Medical College and Research Institute Bengaluru), LD, MB, and AC (PI*, VRDL, All India Institute of Medical Sciences Delhi), BM (PI*, VRDL, Sawai Man Singh Medical College, Jaipur), AJ (PI*, VRDL, King George’s Medical University, Lucknow): coordination, implementation, managing resource availability, and monitoring the testing. PI*: coordination with affiliated health facilities, overseeing sample collection, processing and testing, reporting, documentation, and result entry into portal. ILI-SARI Team (SK, VV, RV, PK, and AD): combo assay preparation, sample collection, processing, data upload, QC and molecular characterization, virus isolation, NAI assay, and training. All authors contributed to the article and approved the submitted version.

## Group members of ILI-SARI Surveillance Team

Sheetal Kadam, ICMR-National Institute of Virology, Pune, India; Veena Vipat, ICMR-National Institute of Virology, Pune, India; Riya Verma, ICMR-National Institute of Virology, Pune, India; Payal Kelkar, ICMR-National Institute of Virology, Pune, India; Anam Dhawlarker, ICMR-National Institute of Virology, Pune, India; Rameshwar Khedekar, ICMR-National Institute of Virology, Pune, India; Vishal Shete, VRDL, All India Institute of Medical Sciences, Nagpur, India; Mangesh Mankar, VRDL, All India Institute of Medical Sciences, Nagpur, India; K. Ismath Jahan, VRDL, Government Mohan Kumaramangalam Medical College, Salem, India; A. Lavanya, VRDL, Government Mohan Kumaramangalam Medical College, Salem, India; Sunitha Pakalapaty, VRDL, Gandhi Medical College, Secunderabad, India; G. Sushma Rajya Lakshmi, VRDL, Gandhi Medical College, Secunderabad, India; Manisha Rani, VRDL, Gandhi Medical College, Secunderabad, India; Narayan Ramamurthy, VRDL, Jawaharlal Institute of Postgraduate Medical Education and Research, Puducherry, India; Sharmila Ferdinamarie, VRDL, Jawaharlal Institute of Postgraduate Medical Education and Research, Puducherry, India; Irfan ul Haq, VRDL, Sher-i-Kashmir Institute of Medical Sciences, Srinagar, India; Suraiya Mir, VRDL, Sher-i-Kashmir Institute of Medical Sciences, Srinagar, India; Harish Bisht, VRDL, Government Medical College, Haldwani, India; Ananya Chatterjee, ICMR-National Institute of Cholera and Enteric Diseases, Kolkata, India; Sutapa Hazra, ICMR-National Institute of Cholera and Enteric Diseases, Kolkata, India; Rudrak Gupta, ICMR-National Institute of Cholera and Enteric Diseases, Kolkata, India; S. Lakshmikanth, ICMR-Regional Medical Research Centre, Port Blair, India; Shivangi Sharma, ICMR-Regional Medical Research Centre, Port Blair, India; Shreedipti Sahoo, ICMR-Regional Medical Research Centre, Bhubaneswar, India; Subhra Subhadra, ICMR-Regional Medical Research Centre, Bhubaneswar, India; Suranjana De, VRDL, Government Medical College, Agartala, India; Ashish Kumar, ICMR-Rajendra Memorial Research Institute of Medical Sciences, Patna, India; V. N. R. Das, ICMR-Rajendra Memorial Research Institute of Medical Sciences, Patna, India; Major Madhukar, ICMR-Rajendra Memorial Research Institute of Medical Sciences, Patna, India; Bhawana Mishra, ICMR-Rajendra Memorial Research Institute of Medical Sciences, Patna, India; Sapana Mishra, VRDL, All India Institute of Medical Sciences, Raipur, India; Kuldeep Sharma, VRDL, All India Institute of Medical Sciences, Raipur, India; Laxmi Rathore, VRDL, Dr. Sampurnanand Medical College, Jodhpur, India; Pragati Pal, VRDL, Dr. Sampurnanand Medical College, Jodhpur, India; Dudekula Chand Basha, VRDL, Sri Venkateswara Institute of Medical Sciences, Tirupati, India; Shubhra Tripathi, VRDL, All India Institute of Medical Sciences, Bhopal, India; Shashwati Nema, VRDL, All India Institute of Medical Sciences, Bhopal, India; Sima Bhatt, VRDL, BJ Medical College, Ahmedabad, India; Hetal Shah, VRDL, BJ Medical College, Ahmedabad, India; Dipa Kinariwala, VRDL, BJ Medical College, Ahmedabad, India; Piyush Patel, VRDL, BJ Medical College, Ahmedabad, India; Chandra Kanta Bhattacharjee, ICMR-Regional Medical Research Centre, Dibrugarh, India; Saritha, VRDL, Government Medical College, Trivandrum, India; Sreelatha, VRDL, Government Medical College, Trivandrum, India; Neethu, VRDL, Government Medical College, Trivandrum, India; Sulaikha Beevi, VRDL, Government Medical College, Trivandrum, India; Jasper Merlin, VRDL, Government Medical College, Trivandrum, India; Arnab Ghosh, VRDL, Post Graduate Institute of Medical Education and Research, Chandigarh, India; Kapil Goyal, VRDL, Post Graduate Institute of Medical Education and Research, Chandigarh, India; Kapil Goel, VRDL, Post Graduate Institute of Medical Education and Research, Chandigarh, India; Ashok Pannu, VRDL, Post Graduate Institute of Medical Education and Research, Chandigarh, India; Jayashree Muralidharan, VRDL, Post Graduate Institute of Medical Education and Research, Chandigarh, India; Ambreen Shaikh, VRDL, Government Medical College, Aurangabad, India; Dhaval Khatri, VRDL, Government Medical College, Aurangabad, India; Ganesh Korhale, VRDL, Government Medical College, Aurangabad, India; Maitrik Dave, VRDL, Government Medical College, Aurangabad, India; Netaji Sapte, VRDL, Government Medical College, Aurangabad, India; Vinod Chavanke, VRDL, Government Medical College, Aurangabad, India; C. P. Anupama, VRDL, King Institute of Preventive Medicine and Research, Chennai, India; Kiruba Ramesh, VRDL, King Institute of Preventive Medicine and Research, Chennai, India; R. Ambica, VRDL, Bangalore Medical College and Research Institute, Bangalore, India; Jyoti Jethani, VRDL, All India Institute of Medical Sciences, New Delhi, India; Shreya Sharma, VRDL, Sawai Man Singh Medical College, Jaipur, India; Haya Khan, VRDL, Sawai Man Singh Medical College, Jaipur, India; Vikas Patel, VRDL, King George’s Medical University, Lucknow, India; Sulekha Verma, VRDL, King George’s Medical University, Lucknow, India; Turya Singh, VRDL, King George’s Medical University, Lucknow, India.
